# From Many Hosts, One Accidental Pathogen: The Diverse Protozoan Hosts of *Legionella*

**DOI:** 10.3389/fcimb.2017.00477

**Published:** 2017-11-30

**Authors:** David K. Boamah, Guangqi Zhou, Alexander W. Ensminger, Tamara J. O'Connor

**Affiliations:** ^1^Department of Biological Chemistry, Johns Hopkins University School of Medicine, Baltimore, MD, United States; ^2^Department of Biochemistry, University of Toronto, Toronto, ON, Canada; ^3^Department of Molecular Genetics, University of Toronto, Toronto, ON, Canada; ^4^Public Health Ontario, Toronto, ON, Canada

**Keywords:** *Legionella*, amoebae, protozoa, host range, environment, *Acanthamoebae*, *Hartmannella*, *Naegleria*

## Abstract

The 1976 outbreak of Legionnaires' disease led to the discovery of the intracellular bacterial pathogen *Legionella pneumophila*. Given their impact on human health, *Legionella* species and the mechanisms responsible for their replication within host cells are often studied in alveolar macrophages, the primary human cell type associated with disease. Despite the potential severity of individual cases of disease, *Legionella* are not spread from person-to-person. Thus, from the pathogen's perspective, interactions with human cells are accidents of time and space—evolutionary dead ends with no impact on *Legionella*'s long-term survival or pathogenic trajectory. To understand *Legionella* as a pathogen is to understand its interaction with its natural hosts: the polyphyletic protozoa, a group of unicellular eukaryotes with a staggering amount of evolutionary diversity. While much remains to be understood about these enigmatic hosts, we summarize the current state of knowledge concerning *Legionella*'s natural host range, the diversity of *Legionella*-protozoa interactions, the factors influencing these interactions, the importance of avoiding the generalization of protozoan-bacterial interactions based on a limited number of model hosts and the central role of protozoa to the biology, evolution, and persistence of *Legionella* in the environment.

## Predator vs. prey: *legionella* and its natural protozoan hosts

In the environment, bacteria are targets of predation by grazing protozoa (Hahn and Höfle, 2001; Molmeret et al., 2005). In response to predation, many bacteria have developed strategies to either avoid predation or survive, and in some cases, replicate within protozoa. As bacteria are destined to encounter a large number of protozoa species in nature, their fitness will be determined by the breadth and diversity of protozoa within which they are able to grow. Though many types of bacteria are able to replicate within protozoa (Greub and Raoult, 2004), this behavior is best characterized in the bacterial pathogen *Legionella*, in particular *Legionella pneumophila*, which will be the major focus of this review.

## *L. pneumophila* in the environment

*L. pneumophila* is ubiquitous in nature (Fliermans, 1996; van Heijnsbergen et al., 2015). While various species of *Legionella* have been isolated from soil and marine environments, freshwater systems serve as the major reservoirs of *L. pneumophila* (Fliermans, 1996; van Heijnsbergen et al., 2015). *L. pneumophila* can exist in a planktonic form however, it is more often found within mixed community biofilms (Mampel et al., 2006). *L. pneumophila* intercalates into existing biofilms (Lau and Ashbolt, 2009; Stewart et al., 2012) where it acquires nutrients by forming synergistic relationships with other members of the biofilm (Tison et al., 1980; Pope et al., 1982; Bohach and Snyder, 1983; Wadowsky and Yee, 1983; Stout et al., 1986; Stewart et al., 2012; Koide et al., 2014). *L. pneumophila* is also capable of surviving in nutrient-poor conditions by necrotrophic growth on dead cell masses (Temmerman et al., 2006). Although, its interactions with other bacteria promote *L. pneumophila* survival in oligotrophic environments, intracellular growth within protozoa is likely the predominant mechanism of *L. pneumophila* proliferation in its natural habitat (Rowbotham, 1980).

## The impact of natural hosts on *legionella* persistence in the environment and pathogenesis

Protozoa function as natural reservoirs of *L. pneumophila* and promote disease in humans. The intracellular environment of the host cell protects *L. pneumophila* from harsh environmental conditions while providing a nutrient rich replicative niche (Greub and Raoult, 2004; Abdel-Nour et al., 2013). The ability of *L. pneumophila* to survive within amoebae also protects the bacteria from killing by water disinfection procedures (Plouffe et al., 1983; King et al., 1988; Kilvington and Price, 1990; Biurrun et al., 1999; Storey et al., 2004; Bouyer et al., 2007; García et al., 2008; Cervero-Aragó et al., 2014, 2015), a reciprocal relationship that also enhances survival of the host (García et al., 2007). As a consequence, *L. pneumophila* are commonly found in man-made potable water supply and distribution systems (Ikedo and Yabuuchi, 1986; Breiman et al., 1990; Yamamoto et al., 1992; Fields et al., 2002; Lasheras et al., 2006; Brousseau et al., 2013; Thomas et al., 2014). Although, there is one reported case of probable human-to-human transmission of *Legionella* (Correia et al., 2016), the vast majority of evidence suggests a non-communicable disease. Instead, human exposure predominantly occurs through the inhalation of contaminated water aerosols (Fields, 1996), which can lead to pneumonic respiratory disease. *L. pneumophila* passaged through amoebae are more virulent in animal models of infection compared to bacteria grown in broth culture (Cirillo et al., 1994, 1999; Barker et al., 1995; Brieland et al., 1996; Garduño et al., 2002). The earliest description of *L. pneumophila*'s interaction with amoebae even proposed that an important route of human infection may be the inhalation of the pathogen in an amoebal-encapsulated state (Rowbotham, 1980). Thus, the interaction of *L. pneumophila* with protozoa is a critical determinant in both the persistence of *Legionella* in environmental and man-made reservoirs, and the incidence and severity of disease.

## The broad host range of *L. pneumophila*

Many bacterial pathogens become highly specialized for growth in one or a small subset of hosts but few are able to grow in multiple hosts. Host jumping has been observed for some pathogens but often comes at a price, the inability to grow in the previous host (Ma et al., 2006). In contrast, *L. pneumophila* exhibits an extensive host range replicating within a diverse array of protozoan hosts that span multiple phyla, from Amoebozoa (amoebae) to Percolozoa (excavates) to Ciliophora (ciliated protozoa) (Rowbotham, 1980; Fields, 1996). The ability to maintain such a broad host range is due to the assembly of a large cohort of genes that allow *L. pneumophila* to adapt to variations between hosts (O'Connor et al., 2011). Moreover, the ability to continually evolve and alter the composition of its virulence gene repertoire allows *L. pneumophila* to adapt to shifts in protozoan populations in their natural habitats (O'Connor et al., 2011). Since the discovery that *L. pneumophila* can survive and replicate within free-living amoeba (Rowbotham, 1980), the relationship between *L. pneumophila* and its protozoa hosts has garnered significant attention, largely due to the important role of protozoa in the epidemiology of this pathogen. In this review, we expand on the early works of Rowbotham and Fields (Rowbotham, 1980, 1986; Fields, 1996) to summarize the current knowledge of the host range of *L. pneumophila* in environmental reservoirs and the factors that impact the outcome of *Legionella*-protozoa interactions.

## The different fates of *L. pneumophila* within protozoan hosts

While *L. pneumophila* has an extensive host range, the fate of the bacterium once it enters the host cell can vary greatly. Several protozoa are able to efficiently deliver *L. pneumophila* to the lysosome for degradation, resulting in the death of the bacterium (Amaro et al., 2015). *L. pneumophila* predation by protozoa does not seem to be restricted to one particular group. While members of the Cercozoa phylum seem to be especially adept at digesting *L. pneumophila* (Amaro et al., 2015), distantly related members of the Amoebozoa phylum (*Cashia limacoides, Vannella platypodia*, and *Vexillifera bacillipedes*) are also efficient at killing *L. pneumophila* (Rowbotham, 1986). In contrast, many protozoa serve as hosts for *L. pneumophila* replication. In these cases, the *Legionella*-protozoa interaction is detrimental to the host: the bacteria multiply to high numbers and then kill the host as they exit the cell (Rowbotham, 1983). Alternatively, *L. pneumophila* can be toxic to the host in the absence of replication, a protist version of food-poisoning (Amaro et al., 2015). *L. pneumophila* within amoebae has been shown to inhibit both amoebae proliferation (Mengue et al., 2016) and chemotactic motility (Simon et al., 2014). The fates of the two organisms are not solely defined by this “it's you or me” relationship, as a number of intermediate outcomes have been observed. In response to extreme stress, amoebae undergo encystation, transforming into a dormant, highly resistant cyst form. While encystation restricts bacterial replication (Rowbotham, 1986; Ohno et al., 2008), *L. pneumophila* is able to survive the encystation process until more favorable conditions arise (Kilvington and Price, 1990; Greub and Raoult, 2003). Similarly, for some *Legionella*-protozoa pairs, *L. pneumophila* is resistant to grazing by the protozoan and thus survives within the host cell but fails to replicate (Smith-Somerville et al., 1991). Alternatively, *L. pneumophila* can be packaged into multi-membrane vesicles that are distinct from the replication vacuole and expelled into the extracellular environment (Rowbotham, 1983; Berk et al., 1998; Hojo et al., 2012; Amaro et al., 2015). The release of *Legionella*-containing pellets has been observed in both the ciliated protozoa *Tetrahymena* spp. (Faulkner et al., 2008; Hojo et al., 2012) and the amoebal hosts *Acanthamoeba castellanii* and *Acanthamoeba astronyxis* (Bouyer et al., 2007; Amaro et al., 2015), and does not appear to coincide with bacterial replication. Whether this process is driven by the bacterium or the host is still unclear. The pellet compartment can protect *L. pneumophila* from environmental stress (Bouyer et al., 2007; Koubar et al., 2011) which would be beneficial during its transition between host cells and thus a potential mechanism to ensure its survival. Consistent with this idea, a functional Type IVb secretion system, a major *L. pneumophila* virulence factor required for lysosome avoidance and intracellular replication, appears to be important for the release of *L. pneumophila* in pellets (Berk et al., 2008). Alternatively, the inability to digest the bacteria may simply trigger a host response that involves bacterial expulsion, as a similar phenomenon is observed with non-pathogenic *Escherichia coli, Bacillus subtilis*, and *Mycobacterium luteus* (Hojo et al., 2012; Denoncourt et al., 2014). Whether *L. pneumophila* resists predation or is expelled in pellets, the host is considered to be only partially restrictive due to the survival of *L. pneumophila* and its potential to transition to other host cells. Indeed, one might speculate that such intermediate host-bacterial interactions (resistance to protozoan predation in the absence of replication) might resemble the first evolutionary step toward becoming an intracellular pathogen.

## Methods for defining protozoan hosts of *legionella*

Protozoan hosts of *Legionella* are defined by two main techniques: co-culture and co-isolation. When combined with microscopy, co-culture techniques allow for the direct visualization of *Legionella* within host cells, and by analyzing infected cells over time, bacterial replication within a particular host provides direct experimental evidence of *Legionella* survival and replication. When combined with plating assays to monitor bacterial numbers, co-culture methods allow bacterial growth rates, maximum growth and the impact of bacterial dose and various external conditions on the interaction to be analyzed. However, while *Legionella* may be able to replicate in a given host under specific laboratory conditions, the experimental system may not reflect conditions encountered in the environment and thus, biologically relevant interactions that commonly occur in nature. Co-isolation studies attempt to address this issue by examining the co-existence of protozoa and *Legionella* in environmental samples. In rare cases, protozoa harboring *Legionella* have been isolated from environmental samples providing direct evidence of their interaction in the environment (Thomas et al., 2006; Hsu et al., 2011; Kao et al., 2013). More commonly, *Legionella* are identified by 16S sequencing of DNA extracts from bacteria isolated by *Legionella*-selective culture methods on bacteriological medium (Salloum et al., 2002; Sheehan et al., 2005) or enrichment through co-culture of environmental samples with amoebae (Pagnier et al., 2008). Protozoa may be identified microscopically by fluorescence *in situ* hybridization (FISH) or the morphological appearance of trophozoites (Jacquier et al., 2013; Muchesa et al., 2014), or by 18S sequencing of DNA extracts following an amoebal enrichment step in which individual isolates are cultured on lawns of bacteria permissive to amoebal grazing (Greub and Raoult, 2004; Delafont et al., 2013; Muchesa et al., 2014). Thus, while most co-isolation studies do not provide direct evidence of *Legionella* growth within the protozoa identified, they can be used to predict environmentally relevant interactions, to substantiate experimental findings from co-culture techniques and are likely to implicate new protozoan species as potential hosts of *Legionella*.

## Experimentally defined protozoan hosts of *L. pneumophila*

**Figure 1:**
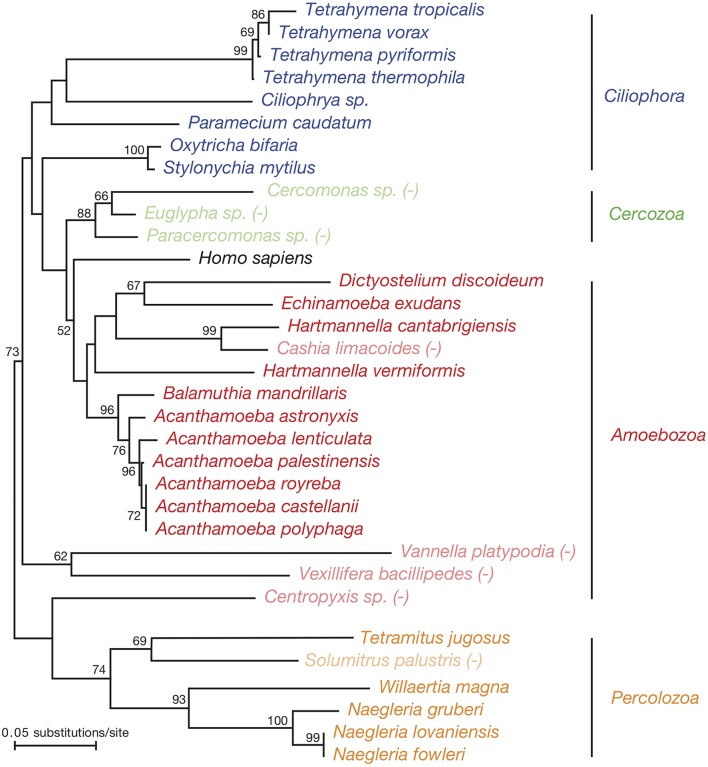
An 18S phylogenetic tree of the experimentally defined hosts of *L. pneumophila*. Evolutionary history was inferred using the Neighbor-Joining method based on an alignment of 18S rRNA sequences. Evolutionary analyses were performed using MEGA7 (Kumar et al., 2016). Restrictive host species that do not support *L. pneumophila* replication or survival are indicated by lighter shading and the annotation “(−)”. Taxonomic designations are based on the classification system outlined in Ruggiero et al. (2015).

The initial discovery that *L. pneumophila* is capable of surviving and replicating in protozoa fostered a number of independent investigations to examine the host range of this bacterium (Table 1). Co-culture methods in combination with various microscopy techniques demonstrated growth of *L. pneumophila* in diverse protozoan hosts encompassing several species of *Acanthamoeba* (*A. castellanii, Acanthamoeba polyphaga*, and *Acanthamoeba palestinensis*), *Hartmannella* (*Vermamoeba vermiformis*, formerly *Hartmannella vermiformis* and *Hartmannella cantabridiensis*) and *Naegleria* (*Naegleria gruberi, Naegleria lovaniensis*, and *Naegleria jadini*) as well as *Tetrahymena pyrofomis, Echinamoeba exudans*, and *Tetramitus jugosus* (formerly *Vahlkampfia jugosus*) (Rowbotham, 1980, 1986; Tyndall and Domingue, 1982; Anand et al., 1983; Barbaree et al., 1986). While the list of hosts was dominated by three particular genera (*Acanthamoeba, Hartmannella*, and *Naegleria*), collectively it represented three different phyla Amoebozoa, Ciliophora, and Percolozoa and amongst them, four distantly related classes of protozoa, Discosea (*Acanthamoebae*), Tubulinea (*Echinamoeba* and *Hartmannella*), Heterolobosea (*Naegleria* and *Tetramitus*), and Oligohymenophorea (*Tetrahymena*) (Figure 1).

**Table 1:** Experimentally defined protozoan hosts of *L. pneumophila*.

**Protozoan species**	**Protozoan strain**	***L. pneumophila* serogroup (Sg): strain**	**Fate of *L. pneumophila***	**Experimental evidence**	**References**
*Acanthamoeba* spp.	AMI137, AMI116, AMI073, AMI191, Humidifier strain	Sg1: Lens	Intracellular multiplication	CFU counting, Phase-contrast microscopy	Rowbotham, 1980; Dupuy et al., 2016
		Sg2: Togus-1			
		Sg3: Bloomington-2			
		Sg5: Cambridge-2			
*Acanthamoeba* sp. 155		Sg1	Intracellular multiplication	CFU counting, Epifluorescence microscopy	Cervero-Aragó et al., 2014, 2015
*Acanthamoeba astronyxis*	Isolate C37C6	Sg1: Philadelphia-1	Live cells are packaged in expelled pellets	Electron microscopy	Marciano-Cabral and Cabral, 2003; Amaro et al., 2015
*Acanthamoeba castellanii*	ATCC® 30234™, CCAP 1534/2, L1501/2A, L501/2A, Neff	Sg1: JR32, Lens, Paris, Philadelphia-1, Philadelphia-2, Pontiac-1	Intracellular multiplication	CFU counting, Electron microscopy	Rowbotham, 1980; Holden et al., 1984; Moffat and Tompkins, 1992; Hilbi et al., 2001; Bouyer et al., 2007; Tyson et al., 2013; Mengue et al., 2016
		Sg2: Togus-1			
		Sg3: Bloomington-2			
		Sg4: Los Angeles			
		Sg6: Oxford-1			
	Neff	Sg5: Dallas 1E	Live cells are packaged in expelled pellets	Electron microscopy	Berk et al., 1998
*Acanthamoeba lenticulata*	PD2	Sg1: AX71, Philadelphia-1, SC94, SC97	Intracellular multiplication	CFU counting	Molmeret et al., 2001
		Sg2: AX2			
		Sg3: AX52, AX54, AX82			
*Acanthamoeba palestinensis*		Sg1	Intracellular multiplication	CFU counting, Electron microscopy, Epifluorescence microscopy, Phase contrast microscopy	Anand et al., 1983; Harf et al., 1997
*Acanthamoeba polyphaga*	Ap-1, L1501/3A, Puschkarew	Sg1: AA100, Corby, Nottingham-8, Leeds 1A SAP, Leeds-4, Lp02, Philadelphia-2, Pontiac-1	Intracellular multiplication	CFU counting, Electron microscopy, Phase-contrast microscopy	Rowbotham, 1980, 1986; Kilvington and Price, 1990; Gao et al., 1997; Buse and Ashbolt, 2011
		Sg2: Oxford-2, Togus-1			
		Sg3: Bloomington-2			
		Sg4: Los Angeles-1			
		Sg5: Cambridge-2			
		Sg6			
		Sg7: Dallas-5, Chicago-8			
		Sg8: York-1, Concord-3			
	Puschkarew	Sg5: Dallas 1E	Intracellular Survival, Live cells are packaged in expelled pellets	CFU counting, Electron microscopy	Berk et al., 1998; Buse and Ashbolt, 2011
*Acanthamoeba royreba*		Sg4: Los Angeles	Intracellular multiplication	Bacteria cell count, Epifluorescence microscopy	Tyndall and Domingue, 1982
*Balamuthia mandrillaris*	CDC-V039	Sg1: JR32, 130b	Intracellular multiplication	CFU counting, Phase-contrast microscopy	Shadrach et al., 2005
*Ciliophrya* sp.		Sg1: Corby	Intracellular survival	Epifluorescence microscopy	Rasch et al., 2016
*Dictyostelium discoideum*	AX2, AX2-214, AX3	Sg1: Benidorm 030E, Corby, Philadelphia-1	Intracellular multiplication	CFU counting, Electron microscopy	Hägele et al., 2000; Solomon et al., 2000
*Echinamoeba exudans*	SH274	Sg1: RI-243	Intracellular multiplication	Electron microscopy	Fields et al., 1989
*Hartmannella cantabrigiensis*		Sg2: PR-1	Intracellular multiplication	Electron microscopy	Rowbotham, 1986
		Sg5: Leeds-10			
		Sg7: Chicago-8, Dallas-5			
		Sg8: York-1			
*Naegleria* spp.	AMI242, AMI117, AMI135, AMI161	Sg1: Lens	Intracellular multiplication	CFU counting	Dupuy et al., 2016
*Naegleria fowleri*	Lee	Sg1: Lp02	Intracellular multiplication	CFU counting, Electron microscopy	Newsome et al., 1985; Buse and Ashbolt, 2011
		Sg3: Bloomington-2			
		Sg6: Chicago-2			
		Sg5: Dallas 1E	Intracellular survival	CFU counting	Buse and Ashbolt, 2011
*Naegleria gruberi*	1518/1E	Sg2: Togus-1	Intracellular multiplication	Phase-contrast microscopy	Rowbotham, 1980
		Sg3: Bloomington-2			
		Sg5: Cambridge-2			
*Naegleria jadini*	B1518/2	Sg2: Togus-1	Intracellular multiplication	Phase-contrast microscopy	Rowbotham, 1980
		Sg3: Bloomington-2			
		Sg5: Cambridge-2			
*Naegleria lovaniensis*	TS	Sg1: Philadelphia-1, 130b	Intracellular multiplication	Confocal microscopy, CFU counting, Bacteria cell count, Epifluorescence microscopy	Tyndall and Domingue, 1982; Declerck et al., 2005; Tyson et al., 2013, 2014
		Sg4: Los Angeles			
*Oxytricha bifaria*		Sg1: Corby	Intracellular survival	Epifluorescence microscopy	Rasch et al., 2016
*Paramecium caudatum*	RB-1	Sg1: Philadelphia-1	Intracellular multiplication	Fluorescence microscopy	Watanabe et al., 2016
*Stylonychia mytilus*		Sg1: Corby	Intracellular survival	Epifluorescence microscopy	Rasch et al., 2016
*Tetrahymena* sp.		Sg1	Intracellular multiplication	CFU counting, Epifluorescence microscopy	Barbaree et al., 1986; Berk et al., 2008
		Sg1: Lp02	Live cells are packaged in expelled pellets	Electron microscopy, Fluorescence microscopy	Berk et al., 2008
*Tetrahymena pyriformis*	No. 500	Sg1: Philadelphia-1, 130b	Intracellular multiplication	CFU counting, Electron microscopy	Fields et al., 1984, 1986; Cianciotto and Fields, 1992
		Sg3: SC-6-C3			
*Tetrahymena thermophila*	Mating type IV	Sg1: Philadelphia-1	Intracellular multiplication	CFU counting, Light microscopy Electron microscopy	Kikuhara et al., 1994
		Sg1: Philadelphia-2	Intracellular survival	CFU counting, Light microscopy Electron microscopy	Kikuhara et al., 1994
	Inbred strain B, SB021	Sg1: JR32	Intracellular multiplication	Electron microscopy; Live cells are packaged in expelled pellets	Hojo et al., 2012
*Tetrahymena tropicalis*		Sg1: Lens, Philadelphia-1	Live cells are packaged in expelled pellets	Electron microscopy	Faulkner et al., 2008; Koubar et al., 2011
*Tetrahymena vorax*	V2S	Sg1: Philadelphia-1	Intracellular survival	Electron microscopy, Fluorescence microscopy	Smith-Somerville et al., 1991
*Tetramitus jugosus^b^* (*Vahlkampfia jugosa*)		Sg1: Leeds 4	Intracellular multiplication	Electron microscopy	Rowbotham, 1986
*Vermamoeba vermiformis^a^* (*Hartmannella vermiformis*)	ATCC® 50256™, CDC-19	Sg1: AA100, Lens, 130b Philadelphia-1, RI-243	Intracellular multiplication	CFU counting, Electron microscopy	Rowbotham, 1986; King et al., 1991; Wadowsky et al., 1995; Abu Kwaik, 1996; Buse and Ashbolt, 2011; Tyson et al., 2013; Dupuy et al., 2016
		Sg5: E-52, E-62			
		Sg6: E-66, E-67			
		Sg1: Lp02	Intracellular survival	CFU counting	Buse and Ashbolt, 2011
		Sg3: Bloomington-2			
		Sg5: Dallas 1E			
		Sg6: Chicago-2,			
		Sg7: Dallas-5, PR-3			
*Willaertia magna*	c2c Maky, T5[S]44, Z503	Sg1: Lens, Paris, Philadelphia-1, 130b	Intracellular multiplication	CFU counting, Electron microscopy	Dey et al., 2009; Tyson et al., 2014

a *Vahlkampfia jugosa has been renamed Tetramitus jugosus (De Jonckheere and Brown, 2005)*.

b *Hartmannella vermiformis has been renamed Vermamoeba vermiformis (Smirnov et al., 2011)*.

Subsequent studies to investigate *L. pneumophila* pathogenesis have progressively expanded the list of protozoan hosts of this bacterium (Table 1 and Figure 1), including additional species of *Acanthamoeba* (*Acanthamoeba lenticulata* and *Acanthamoeba royreba*) and *Naegleria* (*Naegleria fowleri*) as well as more distantly related genera from their respective phyla such as *Dictyostelium discoideum* (Hägele et al., 2000; Solomon et al., 2000) and *Balamuthia mandrillaris* (Amoebozoa) (Shadrach et al., 2005) and *Willertia magna* (Percolozoa) (Dey et al., 2009; Tyson et al., 2014). Similarly, a number of additional ciliated protozoa were identified that were permissive for *L. pneumophila* survival, including *Tetrahymena* spp. (*Tetrahymena tropicalis* and *Tetrahymena vorax*), *Oxytricha bifaria, Stylonychia mytilus, Paramecium caudatum* and a member of the *Ciliophrya* genus, and in one case *L. pneumophila* replication (*Tetrahymena thermophila*), greatly expanding representation from this group (Kikuhara et al., 1994; Rasch et al., 2016; Watanabe et al., 2016). The beneficial interaction of *L. pneumophila* with these organisms appears to be specific as members from each of the representative phyla were also identified that were highly restrictive to *L. pneumophila* survival (Figure 1): *T. vorax* (Ciliophora), *A. astronyxis*, and *Cashia limocoides* (Amoebozoa) and *Solumitrus palustris* (Percolozoa) (Rowbotham, 1986; Smith-Somerville et al., 1991; Amaro et al., 2015). In addition, *L. pneumophila* was unable to grow in *V. platypodia* and *V. bacillipedes* (Rowbotham, 1986), which form a distantly related clade of the Amoebozoa phyla (Figure 1). Similarly, of the members of the Cercozoa phylum examined so far, *Cercomonas* sp., *Euglypha* sp., and *Paracercomonas* sp., all three are restrictive for *L. pneumophila* growth (Amaro et al., 2015; Rasch et al., 2016), suggesting that distinct orders and families within this class may be more restrictive than others. Thus, while the host range of *L. pneumophila* is vast, it does appear to have its limitations.

## Suggested environmental hosts of *L. pneumophila*

Protozoa in both natural and man-made environments can alter the composition of microbial communities by eliminating bacteria through predation or augmenting populations of bacteria that are capable of replicating within these organisms (Yamamoto et al., 1992). Co-isolation techniques have been used to describe the composition of these communities within natural fresh water systems such as hot springs, thermal spas, lakes, ponds, streams, and anthropogenic reservoirs, such as cooling towers, industrial and private water networks and compost facilities. *L. pneumophila* is capable of surviving an array of physical conditions including temperatures ranging from 6 to 63°C (Fliermans et al., 1981). Thermal springs have been of particular interest as they boast characteristically high water temperatures, providing optimal conditions for *L. pneumophila* growth (Hsu et al., 2011; Ji et al., 2014; Rasch et al., 2016). Artificial aquatic reservoirs are of considerable epidemiological significance and typically support higher numbers of bacteria compared to natural water systems (Yamamoto et al., 1992), likely due to higher average water temperatures (Ikedo and Yabuuchi, 1986; Fields et al., 2002; Lasheras et al., 2006). The results of these population level analyses have validated many of the co-culture defined hosts of *L. pneumophila* while identifying several additional potential hosts (Table 2).

**Table 2:** Suggested protozoan hosts of *L. pneumophila*.

**Protozoa**	**Environment source**	**Identification method used**	**References**
*Acanthamoebidae*	Cooling towers	Identified morphologically via microscopy	Yamamoto et al., 1992
*Acanthamoeba* spp.	Compost facilities	Sequence analysis	Conza et al., 2013, 2014
	Cooling towers	Identified morphologically via microscopy	Kurtz et al., 1982
		Sequence analysis	Declerck et al., 2007
	Drinking water systems	Sequence analysis	Marciano-Cabral et al., 2010; Valster et al., 2011; Ji et al., 2014
	Hospital water networks	Identified morphologically via microscopy	Rohr et al., 1998; Steinert et al., 1998
	Industrial water networks	Identified morphologically via microscopy; Sequence analysis	Scheikl et al., 2014
	Natural water systems	Sequence analysis	Declerck et al., 2007; Hsu et al., 2011; Ji et al., 2014
*Acanthamoeba castellanii*	Compost facilities	Sequence analysis	Conza et al., 2013
*Acanthamoeba hatchetti*	Compost facilities	Sequence analysis	Conza et al., 2013, 2014
	Hospital water network	Identified morphologically via microscopy	Breiman et al., 1990
	Natural water systems	Sequence analysis	Hsu et al., 2015
*Acanthamoeba jacobsi*	Natural water systems	Sequence analysis	Hsu et al., 2011
*Acanthamoeba lenticulata*	Compost facilities	Sequence analysis	Conza et al., 2013
*Acanthamoeba palestinensis*	Natural water systems	Sequence analysis	Kao et al., 2013
*Acanthamoeba polyphaga*	Compost facilities	Sequence analysis	Conza et al., 2013, 2014
	Cooling towers	Not specified	Rowbotham, 1986
	Natural water systems	Sequence analysis	Hsu et al., 2009
*Amoebidae*	Cooling towers	Identified morphologically via microscopy	Yamamoto et al., 1992
*Aspidiscidae*	Cooling towers	Identified morphologically via microscopy	Yamamoto et al., 1992
*Bodonidae*	Cooling towers	Identified morphologically via microscopy	Yamamoto et al., 1992
*Cashia limacoides*	Cooling towers	Not specified	Rowbotham, 1986
*Centropyxis* sp.	Natural water systems	Identified morphologically via microscopy	Rasch et al., 2016
*Ciliophrya* sp.	Natural water systems	Identified morphologically via microscopy	Rasch et al., 2016
*Colpodidae*	Cooling towers	Identified morphologically via microscopy	Yamamoto et al., 1992
*Comandonia operculata*	Hospital water network	Identified morphologically via microscopy	Breiman et al., 1990
*Cyclidium* spp.	Cooling towers	Identified morphologically via microscopy	Barbaree et al., 1986
*Diphylleia rotans*	Sewage treatment systems	Sequence analysis	Valster et al., 2010
*Echinamoeba* spp.	Hospital water networks	Identified morphologically via microscopy	Rohr et al., 1998
*Echinamoeba exudans*	Drinking water systems	Sequence analysis	Valster et al., 2011
	Hospital water networks	Identified morphologically via microscopy	Fields et al., 1989
*Echinamoeba thermarum*	Drinking water systems	Sequence analysis	Valster et al., 2011
	Cooling towers	Sequence analysis	Valster et al., 2010
*Euglypha* sp.	Natural water systems	Identified morphologically via microscopy	Rasch et al., 2016
*Filamoeba nolandi*	Hospital water networks	Identified morphologically via microscopy	Breiman et al., 1990
*Flamella balnearia*	Compost facilities	Sequence analysis	Conza et al., 2013
*Hartmannellidae*	Cooling towers	Identified morphologically via microscopy	Yamamoto et al., 1992
*Hartmannella* spp.	Cooling towers	Sequence analysis	Declerck et al., 2007
		Identified morphologically via microscopy	Kurtz et al., 1982
	Hospital water networks	Identified morphologically via microscopy	Fields et al., 1989; Breiman et al., 1990; Nahapetian et al., 1991
	Natural water systems	FISH; Identified morphologically via microscopy	Zbikowska et al., 2014
		Sequence analysis	Declerck et al., 2007
*Hartmannella cantabrigiensis*	Hospital water networks	Identified morphologically via microscopy	Rowbotham, 1986; Fields et al., 1989
*Learamoeba waccamawenis*	Compost facilities	Sequence analysis	Conza et al., 2013, 2014
*Mayorella* spp.	Hospital water networks	Identified morphologically via microscopy	Steinert et al., 1998
*Naegleria* spp.	Cooling towers	Identified morphologically via microscopy	Barbaree et al., 1986
		Sequence analysis	Declerck et al., 2007
	Compost facilities	Sequence analysis	Conza et al., 2013, 2014
	Drinking water systems	Sequence analysis	Marciano-Cabral et al., 2010; Ji et al., 2014
	Hospital water networks	Identified morphologically via microscopy	Nahapetian et al., 1991; Rohr et al., 1998
	Industrial water networks	Identified morphologically via microscopy	Scheikl et al., 2014
	Natural water systems	Sequence analysis	Declerck et al., 2007; Hsu et al., 2011; Ji et al., 2014
		FISH; Identified morphologically via microscopy	Zbikowska et al., 2014
*Naegleria australiensis*	Compost facilities	Sequence analysis	Conza et al., 2013
	Natural water systems	Sequence analysis	Huang and Hsu, 2010
*Naegleria fowleri*	Thermal saline bath	FISH; Identified morphologically via microscopy	Zbikowska et al., 2013
	Natural water systems	FISH; Identified morphologically via microscopy	Zbikowska et al., 2014
*Naegleria gruberi*	Compost facilities	Sequence analysis	Conza et al., 2013
	Natural water systems	Sequence analysis	Hsu et al., 2015
*Naegleria lovaniensis*	Natural water systems	Sequence analysis	Huang and Hsu, 2010; Kao et al., 2013
*Naegleria pagei*	Natural water systems	Sequence analysis	Huang and Hsu, 2010
*Neoparamoeba* spp.	Drinking water systems	Sequence analysis	Valster et al., 2011
	Natural water systems	Sequence analysis	Valster et al., 2010
*Oxytricha bifaria*	Natural water systems	Identified morphologically via microscopy	Rasch et al., 2016
*Paravahlkampfia ustiana*^a^ (*Vahlkampfia ustiana*)	Hospital water networks	Identified morphologically via microscopy	Breiman et al., 1990
*Pleuronematidae*	Cooling towers	Identified morphologically via microscopy	Yamamoto et al., 1992
*Rhinosporidium* sp.	Tap water system	Sequence analysis	Valster et al., 2010
*Saccamoeba* spp.	Hospital water networks	Identified morphologically via microscopy	Rohr et al., 1998
*Singhamoeba horticola*	Compost facilities	Sequence analysis	Conza et al., 2013, 2014
*Stenamoeba* spp.	Compost facilities	Sequence analysis	Conza et al., 2013, 2014
*Stenamoeba limacina*	Compost facilities	Sequence analysis	Conza et al., 2014
*Stylonychia mytilus*	Natural water systems	Identified morphologically via microscopy	Rasch et al., 2016
*Tetrahymenidae*	Cooling towers	Identified morphologically via microscopy	Yamamoto et al., 1992
*Tetrahymena* spp.	Cooling towers	Identified morphologically via microscopy	Barbaree et al., 1986
*Tetramitu*s spp.	Compost facilities	Sequence analysis	Conza et al., 2013
*Tetramitus enterica*^b^ (*Vahlkampfia enterica*)	Compost facilities	Sequence analysis	Conza et al., 2013
*Vahlkampfia* spp.	Compost facilities	Sequence analysis	Conza et al., 2014
	Cooling towers	Sequence analysis	Declerck et al., 2007
	Drinking water systems	Sequence analysis	Marciano-Cabral et al., 2010
	Hospital water networks	Identified morphologically via microscopy	Breiman et al., 1990; Rohr et al., 1998; Steinert et al., 1998
	Natural water systems	Sequence analysis	Declerck et al., 2007; Hsu et al., 2011
*Vahlkampfia avara*	Compost facilities	Sequence analysis	Conza et al., 2013, 2014
*Vannella* spp.	Hospital water networks	Identified morphologically via microscopy	Rohr et al., 1998
*Vannella platypodia*	Cooling towers	Not specified	Rowbotham, 1986
*Vermamoeba vermiformis*^c^ (*Hartmannella vermiformis*)	Compost facilities	Sequence analysis	Conza et al., 2013, 2014
	Drinking water systems	Sequence analysis	Valster et al., 2011; Ji et al., 2014
	Hospital water networks	Identified morphologically via microscopy	Rowbotham, 1986; Fields et al., 1989; Breiman et al., 1990; Rohr et al., 1998
		Sequence analysis	Thomas et al., 2006
	Industrial water networks	Identified morphologically via microscopy	Scheikl et al., 2014
	Natural water systems	Sequence analysis	Hsu et al., 2011, 2015; Ji et al., 2014
		Sequence analysis	Kao et al., 2013
		Sequence analysis	Valster et al., 2010
	Tap water systems	Sequence analysis	Valster et al., 2010
*Vexillifera bacillipedes*	Cooling towers	Not specified	Rowbotham, 1986
*Vorticellidae*	Cooling towers	Identified morphologically via microscopy	Yamamoto et al., 1992
*Willaertia* spp.	Cooling towers	Sequence analysis	Declerck et al., 2007
	Natural water systems	Sequence analysis	Declerck et al., 2007
*Willaertia magna*	Compost facilities	Sequence analysis	Conza et al., 2013

a *Vahlkampfia ustiana has been renamed Paravahlkampfia ustiana*.

b *Vahlkampfia enterica has been renamed Tetramitus enterica*.

c *Hartmannella vermiformis has been renamed Vermamoeba vermiformis (Smirnov et al., 2011)*.

There is tremendous concordance between co-culture-confirmed *Legionella*-protozoa interactions and the results of co-isolation studies (Tables 1, 2). With the exception of *Balamuthia* and *Dictyostelium*, all protozoan genera shown to support intracellular growth in laboratory co-culture studies reside with *L. pneumophila* in the environment (Table 2). While this is not surprising for *Acanthamoeba, Hartmannella*, and *Naegleria*, as these are some of the most abundant protozoa in nature, in many cases co-isolation studies identified the same species of these genera. In particular, three of the protozoa identified, *A. palestinensis, N. lovaniensis*, and *V. vermiformis* that had been shown to support *L. pneumophila* replication in co-culture experiments (Anand et al., 1983; Rowbotham, 1986; Declerck et al., 2005; Thomas et al., 2006) were isolated from water samples harboring *L. pneumophila* (Kao et al., 2013). Similarly, amoebal enrichment assays resulted in the isolation of *Acanthamoeba jacobsi* harboring *L. pneumophila* directly from a thermal spring water sample (Hsu et al., 2011). These results identify *A. jacobsi* as a new host of *L. pneumophila* and provide direct evidence of an interaction between *L. pneumophila* and these four protozoan hosts in the environment. The lack of co-isolation of *L. pneumophila* with either *Balamuthia* or *Dictyostelium* species is likely because these protozoa are typically found in soil and the majority of samples analyzed were isolated from aquatic environments (Dunnebacke et al., 2004; Vadell and Cavender, 2007). The high degree of correlation between the co-culture and co-isolation studies supports the role of these organisms as natural hosts of *L. pneumophila* in environmental reservoirs.

Co-isolation studies predict a number of additional phyla and classes of protozoa may support *L. pneumophila* survival or growth (Table 2). In addition to the Amoebozoa, Ciliophora, and Percolozoa phyla, protozoa from Apusozoa (*Diphylleia rotans*), Cercozoa (*Euglypha* sp.), Euglenozoa (*Bodonidae* sp.), and Opsithokonta (*Rhinosporidium* sp.) were identified. Two additional classes of protozoa from previously identified phyla are also represented, Variosea (*Flamella balnearia*) and Oligohymenophorea with representatives encompassing four different families spanning three orders within this group. For those classes of protozoa already identified as hosts by co-culture experiments, three additional orders, Thecamoebida (*Stenamoeba limacina*), Arcellinida (*Centropyxis* sp.), and Sporadotricina (*Aspidiscidae* family) and five genera (*Comandonia operculata, C. limacoides, Paravahlkampfia ustiana, Learamoeba waccamawenis*, and *Singhamoeba horticola*) were identified. Finally, of the known hosts of *L. pneumophila* from co-culture experiments, additional species of *Acanthamoeba* (*A. jacobsi*), *Naegleria* (*Naegleria pagei* and *Naegleria australiensis*), *Tetramitus* (*Tetramius enterica*), and *Vahlkampfia* (*Valkampfia avara*) were also isolated. Combined, co-isolation and co-culture experiments represent 7 of the 8 phyla of the protozoa kingdom, 12 of the 41 classes within these phyla and 21 of the 82 defined orders, demonstrating the tremendous diversity amongst *L. pneumophila* hosts.

Protozoa more commonly found associated with *L. pneumophila* in environmental reservoirs may indicate that they are more likely to be true hosts of the bacterium. While the *Acanthamoeba* spp., *Naegleria* spp., *Vahlkampfia* spp., and *Hartmannella* spp. (including *Vermamoeba vermifomis*) are commonly found in multiple sources (Table 2), particular protozoa appear to co-reside with *L. pneumophila* in more than one environmental sample (Table 2). *A*. *hatchetti, A*. *polyphaga, H*. *cantabrigensis, N*. *fowleri, N*. *lovaniensis, Neoparamoeabe* sp., and *Willertia* sp. have been isolated from both natural and man-made water sources (Table 2), suggesting that these protozoa may function as hosts of *L. pneumophila* in both natural reservoirs and potable water. Both *E. exudans* and *Echinamoeba thermarum* have been identified in more than one potable water sample (Table 2), suggesting these amoebae may play more prominent roles in the epidemiology of *L. pneumophila*. A higher incidence of specific protozoa with *L. pneumophila* may indicate a stronger likelihood that these protozoa are responsible for the persistence of *L. pneumophila* in environmental reservoirs.

Not all protozoa species isolated from the same environmental source are hosts of *L. pneumophila*. Of several species of free-living amoeba collected from a cooling tower, only *A. polyphaga* supported intracellular growth of *L. pneumophila* whereas *L. pneumophila* failed to replicate within *C. limacoides, V. platypodia*, and *V. bacillipedes* (Rowbotham, 1986). Similarly, of several ciliated protozoa species in biofilm samples isolated from a thermal spa, *L. pneumophila* was able to infect *Ciliophrya* sp., *O. bifaria*, and *S. mytilus*, but no intracellular bacteria were detected within *Euglypha* sp. or *Centropyxis* sp. (Rasch et al., 2016). Thus, *L. pneumophila* is able to persist in environments comprised of both *L. pneumophila*-restrictive and permissive protozoan hosts. The relative abundancy of *L. pneumophila* in different environmental niches may reflect mixed populations of these two types of protozoa. Alternatively, in some circumstances *L. pneumophila* may deplete entire populations of permissive hosts, enriching for resistant species of protozoa that remain. Thus, the absence of certain types of protozoa may not necessarily rule them out as contributors to *L. pneumophila* growth and persistence in the environment.

The distribution of protozoa between the types of water sources examined (natural water reservoirs, cooling towers, potable water distribution system, and compost sites; Table 2) was relatively uniform with a few notable exceptions. Amoebozoa and Percolozoa, making up the majority of the protozoa identified, were found in all water sources. Amoebozoa were more predominant in cooling towers and potable water systems. The lower abundance of Percolozoa in cooling towers coincided with a higher abundance of Ciliophora (ciliated protozoa) whereas in potable water, an enrichment in organisms from the Tubulinea class of Amoebozoa, in particular *Echinamoeba* was observed. In contrast, fewer members of the Discosea class were reported and in particular, no members of the Centramoebida order despite their presence in all other sites. The perseverance of *L. pneumophila* within various water environments despite variation in the protozoa composition demonstrates the highly adaptive nature of this bacterium to fluctuations in host population dynamics.

## Metagenomics

Although co-isolation studies provide valuable insights into the microbial communities that support *L. pneumophila*, these methods cannot adequately define the full diversity of these communities (Kunin et al., 2008). While enrichment steps are often necessary to identify low abundance organisms, they create experimental bottlenecks and biases by selecting against protozoa that cannot be cultured using standard protocols (Hugenholtz and Tyson, 2008; Gomez-Alvarez et al., 2012) and *Legionella* isolates with host specificities that do not overlap with amoebal species commonly used in these techniques (Evstigneeva et al., 2009). Metagenome-based analyses may circumvent the limitations inherent to culture-based approaches and provide a more comprehensive, unbiased profile of these communities (Hugenholtz and Tyson, 2008; Gomez-Alvarez et al., 2009). For example, metagenomic studies of samples from three separate watersheds showed both a high level of diversity in the population of *Legionella* (encompassing 15 different species) and a correlation between the levels of Amoebozoa present in the water and the abundance of *Legionella* isolates (Peabody et al., 2017). Monitoring the abundance of *Legionella, Hartmannella*, and *Naegleria* from two environmental water sources over the course of a standard water purification procedure suggested a correlation between the abundance of *Legionella* and *Naegleria*, but not *Hartmannella* (Lin et al., 2014). In general however, metagenomics studies have been somewhat difficult to interpret. Often individual sites are dominated by one or a few amoebal species and the relative abundance of *L. pneumophila* is extremely low compared to other bacteria (Liu et al., 2012; Delafont et al., 2013): these features make it difficult to correlate the presence of *L. pneumophila* with specific protozoa. As the sensitivity and depth of metagenomics analysis improves, metagenomics will most certainly be a source of tremendous insight into the full repertoire of protozoan hosts of *L. pneumophila*.

## Factors affecting the outcome of *legionella*-protozoa interactions

The outcome of the interaction between *L. pneumophila* and protozoa can be influenced by a number of factors; the identity of the host cell, variations in the predatory behavior or feeding preferences of the host, the strain or species of the bacterium, the relative abundance of the two organisms, the external environment, and other microorganisms.

The identity of the host cell can greatly impact the outcome of the infection. While some hosts are permissive for *L. pneumophila* replication, others are restrictive, either impeding bacterial growth or in extreme cases, survival (Amaro et al., 2015). The maximum amount and rate of *L. pneumophila* growth between hosts can vary significantly (Declerck et al., 2005). For example, *L. pneumophila* can achieve up to 10,000-fold growth in *A. castellanii* but only 10-fold growth in *N. lovaniensis* over the same time period (Declerck et al., 2005). Similarly, *L. pneumophila* strain Paris grows robustly in *A. castellanii* and *V. vermiformis* but is defective for growth in *W. magna* (Dey et al., 2009). Moreover, the differential growth of *L. pneumophila* Paris varies between different strains of *W*. *magna*, with robust growth in strain T5[S]44 (Tyson et al., 2014) but failure to grow in strains c2c Maky or Z502 (Dey et al., 2009). Thus, some hosts are more optimal than others for *L. pneumophila* survival and replication.

The predatory behavior and feeding preferences of the host can also influence *Legionella*-protozoa interactions. For example, the *L. pneumophila* auto-inducer LAI-1 disrupts chemotactic migration of *D*. *discoideum* (Simon et al., 2015) and promotes *L. pneumophila* uptake in both *D*. *discoideum* and *A*. *castellanii* (Tiaden et al., 2010). By restricting amoebal movement, *L. pneumophila* may localize feeding to the site of the bacteria—such modulation may also enrich for specific types of amoebae that support *L. pneumophila* replication. The LAI-1 biosynthesis genes are not conserved in all *Legionella* species (Burstein et al., 2016) suggesting that individual species may differentially promote their interaction with amoebae or do so via different mechanisms. Consistent with this idea, the host cell receptors that mediate *L. pneumophila* adhesion to *V*. *vermiformis, A*. *castellanii, A*. *polyphaga*, and *N*. *lovaniensis* and the underlying mechanisms governing bacterial uptake vary between these amoebal hosts (Venkataraman et al., 1997; Harb et al., 1998; Declerck et al., 2005, 2007). As a consequence, bacterial uptake can vary between protozoa. Indeed, *A*. *castellanii* has been shown to ingest *L. pneumophila* with much greater efficiency than *N*. *lovaniensis* (Declerck et al., 2005). Variations in sensing, targeting, adhesion and phagocytosis of bacteria can influence the affinity, specificity, frequency and duration with which *L. pneumophila* interacts with specific protozoa and thus, the impact of their cohabitation on the persistence of *L. pneumophila* in environmental reservoirs.

The genetic composition of the bacterium can greatly impact its fate within the host cell, as the survival and replication of different strains and species of *Legionella* can vary dramatically. Despite the growth defect of *L. pneumophila* Paris in *Willertia magna*, both the *L. pneumophila* Philadelphia-1, Lens and 130b strains are able to replicate in this amoebal host (Dey et al., 2009; Tyson et al., 2014). Similarly, comparisons between clinical and environmental isolates of *L. pneumophila* showed that while one clinical isolate was highly adept at growing in *A. lenticulata* another was severely defective and the relative amounts of replication of the environmental isolates in this host were somewhere in between (Molmeret et al., 2001). Similar differences are observed between species of *Legionella*. While *L. pneumophila, Legionella steelei, Legionella dumoffii*, and *Legionella norrlandica* are able to grow within *A*. *castellanii*, several other species including *Legionella longbeachae, Legionella jordanis*, and *Legionella anisa* are unable to do so (Neumeister et al., 1997; Edelstein et al., 2012; Rizzardi et al., 2014). Thus, the fate of both the bacterium and the host cell is greatly determined by the inherent properties of each organism.

The outcome of a *Legionella*-protozoa interaction is not only influenced by their respective identities but the relative abundance of each organism. For instance, when *L. pneumophila* is present at low levels they are digested for nutrients by *Tetrahymena* sp. but when the bacteria reach a threshold concentration, they are packaged into vesicles and secreted in pellets (Berk et al., 2008; Hojo et al., 2012). The greater the number of bacteria present, the greater the production and secretion of these bacterial pellets. Similar packaging and secretion of other types of bacteria (Denoncourt et al., 2014) suggests this may be a mechanism by which protozoa compensate for over-eating, or stock-pile food (Hojo et al., 2012).

The external environment can have a profound effect on *Legionella*-protozoa interactions. For example, temperature can greatly impact the intracellular fate of *L. pneumophila*. Although, intracellular replication of *L. pneumophila* in *A. castellanii* occurs at a range of temperatures (Rowbotham, 1981), intracellular growth is significantly reduced at lower temperatures (Ohno et al., 2008). Within more restrictive hosts, such as *A*. *polyphaga*, intracellular replication only occurs at higher temperatures whereas below 25°C, *L. pneumophila* is readily consumed (Nagington and Smith, 1980). In contrast, in *Tetrahymena* spp. *L. pneumophila* exhibits robust intracellular growth at 35°C (Fields et al., 1984; Barbaree et al., 1986; Kikuhara et al., 1994) but at lower temperatures, *L. pneumophila* is packaged into vesicles and secreted into the environment (Faulkner et al., 2008; Koubar et al., 2011). The factors affecting intracellular growth of *L. pneumophila* are not mutually exclusive, as different combinations of the strain of *L. pneumophila*, the host cell type and temperature can significantly alter intracellular growth of the bacterium (Buse and Ashbolt, 2011).

Much of the research examining *Legionella*-protozoa interactions has focused on specific bacterial-host pairings, which cannot address the impact of other organisms on these interactions. *L. pneumophila* naturally inhabits complex microbial communities, which could have both positive and negative impacts on *L. pneumophila* survival and population dynamics. For example, *A*. *castellanii* harboring the endosymbiont *Neochlamydia S13* are unable to support *L. pneumophila* replication despite efficient uptake and lack of degradation in the lysosome (Ishida et al., 2014). The impact of *Neochlamydia S13* on *L. pneumophila* replication is specific because *L. pneumophila* is able to replicate in *A*. *castellanii* infected with the endosymbiont *Protochlamydia R18*. Moreover, curing *A*. *castellanii* of *Neochlamyida S13* restores intracellular growth of *L. pneumophila*, suggesting that the presence of the endosymbiont renders *A*. *castellani* resistant to *L. pneumophila* pathogenesis. In contrast, *L. pneumophila* has been shown to promote the intracellular growth of *Brucella neotomae* when the two pathogens share the same vacuole (Kang and Kirby, 2017). While sharing resources does not appear to affect *L. pneumophila*, it is conceivable that *L. pneumophila* may similarly benefit from the activities of other bacteria when it finds itself in more restrictive protozoan hosts.

## Future directions

A critical challenge in understanding the molecular mechanisms of *L. pneumophila* pathogenesis, evolution and environmental persistence is the staggering diversity of the protozoan hosts that support *L. pneumophila* replication. Indeed, such diversity is thought to be responsible for shaping *L. pneumophila* into a generalist pathogen with a broad host range—a feature clearly important for pathogenesis in humans. Rather than having a single, defined “natural host,” *L. pneumophila* wanders from host to host and is constantly shaped by these disparate interactions. Such a lifestyle is a challenge for researchers studying these bacteria: (1) many protozoa remain poorly characterized, difficult to culture, and/or unsequenced; (2) the shear diversity of protozoa and complexity of natural interactions makes experimental analysis of phenotypes under “physiologically relevant” conditions extremely daunting (which hosts should be used and under what chemical and physical conditions should the interaction be studied?); and (3) how can non-binary interactions with mixed bacterial and host populations be examined in a reproducible and informative fashion? Given the importance of protozoa to *L. pneumophila* biology (and pathogen evolution in general), we strongly advocate efforts for the sequencing and detailed study of these organisms. While it is enticing to retreat to the comfort of studying *Legionella*-host interactions in mammalian macrophages and perhaps one or two model protozoa, an exciting, informative, frustrating, and messy reality remains largely unexplored. Perhaps once the diversity of bacterial-protozoan behaviors is better understood, a panel of model hosts could be chosen not based on ease of culture, but instead to capture the greatest breadth of this diversity.

## Author contributions

TO, DB, AE, and GZ wrote the manuscript. GZ and AE generated the phylogenetic tree.

### Conflict of interest statement

The authors declare that the research was conducted in the absence of any commercial or financial relationships that could be construed as a potential conflict of interest.
